# Supplementary material for: CTRP3 and serum triglycerides in children aged 7-10 years
Source: PLoS One. 2020 Dec 3;15(12):e0241813. doi: 10.1371/journal.pone.0241813 (PMC7714231; doi:10.1371/journal.pone.0241813)
Supplement: S1 Fig — (DOCX) [file pone.0241813.s002.docx]

S1 Fig: CTRP3 was not detectable in the LMW fraction


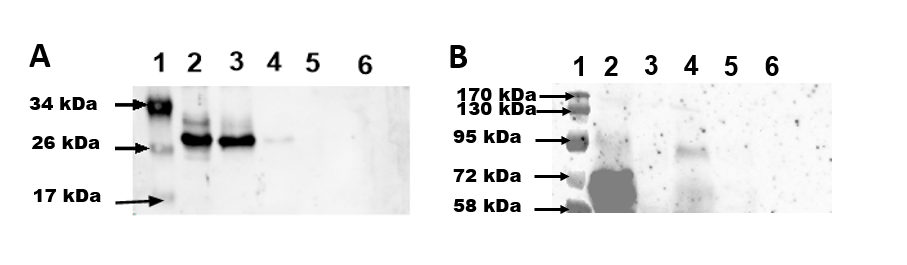


Lane 1: protein ladder (Fisher Cat#BP3606); Lane 2: Total serum; lane 3: HMW fraction; Lane 4: MMW fraction; Lane 5: LMW fraction; lane 6: final flow through.

Methods for Figure S1: A Pooled sample was created from an aliquot of serum collected from 10 randomly selected samples from the study population and diluted in phosphate buffered saline. Samples were separated by size using centrifugal separation (Sartorius™ Vivaspin™; VS0151) according to manufactures directions. The flow through contained all the molecules below 300 kDa in size and the concentrate contained only those molecules equal to or above 300 kDa in size representing the High molecular weight (HMW) fraction. The flow through was then subjected to 100 kDa centrifugal separation (Millipore, #UFC510024). The concentrate from the second round of centrifugal separation contained molecules larger than 100 kDa but less than 300 kDa or the middle molecule weight (MMW) fraction. The flow through from the second round of centrifugal separation, containing the proteins and isomers less than 100 kDa or Low molecule weight (LMW) fraction, was concentrated using a 10kDa cutoff filter (Pierce, #88513). An aliquot of undiluted total pooled serum was diluted 1:20 (v/v) in Laemmli buffer (2% SDS, 5% 2-mercaptoethanol, 5 mM DTT, 10% glycerol, 0.002% bromophenol blue, 0.062 M Tris HCL, pH 6.8). The concentrates containing the HMW or MMW fractions were also diluted to 1:20 (v/v) in Laemmli buffer. The concentrate containing the LMW fraction and the final flow through were diluted 1:2 (v/v) in 2x Laemmli buffer. The samples were then denatured by heating 100°C, and then separated by routine SDS-page electrophoresis and immunoblot. Figure A was incubated with CTRP3 antibody (~28 kDa, R and D Systems Cat# AF7925), figure B was incubated with Transferrin antibody (~77 kDa, R and D Systems Cat# MAB5746),
