# Supplementary material for: CTRP3 and serum triglycerides in children aged 7-10 years
Source: PLoS One. 2020 Dec 3;15(12):e0241813. doi: 10.1371/journal.pone.0241813 (PMC7714231; doi:10.1371/journal.pone.0241813)
Supplement: S1 Table — (DOCX) [file pone.0241813.s003.docx]

S1 Table: Spearman's rank-order correlation coefficient for Total CTRP3 and other metabolic parameters

|  | R^2^ | p value |
| --- | --- | --- |
| MMW CTRP3 (ng/mL) | 0.178 | 0.167 |
| HMW CTRP3 (ng/mL) | 0.537 | **<0.001** |
| Adiponectin (ug/mL) | 0.242 | 0.060 |
| C-Peptide (pg/mL) | 0.219 | 0.087 |
| Ghrelin (pg/mL) | 0.011 | 0.933 |
| Glucagon (pg/mL) | 0.125 | 0.332 |
| Leptin (pg/mL) | 0.026 | 0.844 |
| IL-6 (pg/mL) | 0.063 | 0.626 |
| TNF (pg/mL) | -0.004 | 0.976 |
| C-Reactive Protein (pg/mL) | -0.072 | 0.577 |
| Insulin (pg/mL) | 0.138 | 0.289 |
| Triglycerides (mg/dL) | 0.046 | 0.724 |
| Total Cholesterol (mg/dL) | 0.270 | **0.034** |
| HDL (mg/dL) | 0.271 | **0.033** |
| LDL (mg/dL) | 0.017 | 0.212 |
| VLDL (mg/dL) | 0.017 | 0.898 |
| BMI (kg/m^2^) | -0.068 | 0.598 |

The Spearman's rank-order correlation coefficient and p-values are reported for all values (n=62). Abbreviations: MMW, middle molecular weight; HMW, high molecule weight; IL-6, Interleukin 6; TNF, tumor necrosis factor; HDL, high-density lipoproteins; LDL, low-density lipoproteins; VLDL, very low density lipoprotein; BMI, Body mass index (kg/m^2^).
