# Supplementary material for: CTRP3 and serum triglycerides in children aged 7-10 years
Source: PLoS One. 2020 Dec 3;15(12):e0241813. doi: 10.1371/journal.pone.0241813 (PMC7714231; doi:10.1371/journal.pone.0241813)
Supplement: S2 Table — (DOCX) [file pone.0241813.s004.docx]

S2 Table: Spearman's rank-order correlation coefficient for HMW CTRP3 and other metabolic parameters

|  | R^2^ | p value |
| --- | --- | --- |
| Total CTRP3 (ng/mL) | 0.537 | **<0.001** |
| MMW CTRP3 (ng/mL) | <0.001 | 0.999 |
| Adiponectin (ug/mL) | 0.048 | 0.710 |
| C-Peptide (pg/mL) | -0.069 | 0.596 |
| Ghrelin (pg/mL) | -0.076 | 0.558 |
| Glucagon (pg/mL) | -0.056 | 0.666 |
| Leptin (pg/mL) | -0.045 | 0.728 |
| IL-6 (pg/mL) | 0.007 | 0.958 |
| TNF (pg/mL) | -0.194 | 0.131 |
| C-Reactive Protein (pg/mL) | 0.017 | 0.897 |
| Insulin (pg/mL) | 0.081 | 0.537 |
| Triglycerides (mg/dL) | 0.077 | 0.553 |
| Total Cholesterol (mg/dL) | 0.017 | 0.898 |
| HDL (mg/dL) | 0.214 | 0.095 |
| LDL (mg/dL) | -0.083 | 0.521 |
| VLDL (mg/dL) | 0.052 | 0.692 |
| BMI (kg/m^2^) | -0.151 | 0.240 |

The Spearman's rank-order correlation coefficient and p-values are reported for all values (n=62). Abbreviations: MMW, middle molecular weight; HMW, high molecule weight; IL-6, Interleukin 6; TNF, tumor necrosis factor; HDL, high-density lipoproteins; LDL, low-density lipoproteins; VLDL, very low density lipoprotein; BMI, Body mass index (kg/m^2^).
