# Supplementary material for: CTRP3 and serum triglycerides in children aged 7-10 years
Source: PLoS One. 2020 Dec 3;15(12):e0241813. doi: 10.1371/journal.pone.0241813 (PMC7714231; doi:10.1371/journal.pone.0241813)
Supplement: S3 Table — (DOCX) [file pone.0241813.s005.docx]

S3 Table: Spearman's rank-order correlation coefficient for MMW CTRP3 and other metabolic parameters

|  | R^2^ | p value |
| --- | --- | --- |
| Total CTRP3 (ng/mL) | 0.178 | 0.166 |
| HMW CTRP3 (ng/mL) | <0.001 | 0.999 |
| Adiponectin (ug/mL) | **0.262** | **0.039** |
| C-Peptide (pg/mL) | -0.127 | 0.3240 |
| Ghrelin (pg/mL) | 0.217 | 0.090 |
| Glucagon (pg/mL) | -0.021 | 0.873 |
| Leptin (pg/mL) | -0.098 | 0.450 |
| IL-6 (pg/mL) | 0.174 | 0.176 |
| TNF (pg/mL) | 0.142 | 0.270 |
| C-Reactive Protein (pg/mL) | -0.146 | 0.257 |
| Insulin (pg/mL) | **-0.289** | **0.024** |
| Triglycerides (mg/dL) | **-0.826** | **0.000** |
| Total Cholesterol (mg/dL) | -0.232 | 0.070 |
| HDL (mg/dL) | 0.231 | 0.071 |
| LDL (mg/dL) | -0.035 | 0.787 |
| VLDL (mg/dL) | **-0.827** | **0.000** |
| BMI (kg/m2) | **-0.357** | **0.004** |

The Spearman's rank-order correlation coefficient and p-values are reported for all values (n=62). Abbreviations: MMW, middle molecular weight; HMW, high molecule weight; IL-6, Interleukin 6; TNF, tumor necrosis factor; HDL, high-density lipoproteins; LDL, low-density lipoproteins; VLDL, very low density lipoprotein; BMI, Body mass index (kg/m^2^).
