# Supplementary material for: CTRP3 and serum triglycerides in children aged 7-10 years
Source: PLoS One. 2020 Dec 3;15(12):e0241813. doi: 10.1371/journal.pone.0241813 (PMC7714231; doi:10.1371/journal.pone.0241813)
Supplement: Attachment — Submitted filename: PONE-D-20-22639_reviewer comments.docx [file pone.0241813.s006.docx]

PLoS ONE reviewer comments

Summary: This study measured CTRP3 HMW and presumed MMW circulating levels from blood samples taken from pediatric donors. Key finding was that circulating triglycerides and VLDL levels negatively correlated with MMW CTRP3, while HMW CTRP3 did not show any statistically significant correlation with the measured parameters.

Significance: The circulating levels of CTRP3 oligomeric complexes have previously been examined in an adult cohort with or without Type 2 Diabetes Mellitus. This is a similar study on a pediatric cohort. While the reported parameters are similar between the two studies, CTRP3 levels have not previously been examined in a pediatric demographic, and this study provides novel information relevant to the growing epidemic of childhood obesity and type 2 diabetes.

The manuscript is clearly written except for some typographical errors throughout the manuscript and a few run-on sentences. Authors should carefully review the manuscript for typographical and grammatical errors. Appropriate statistical analyses have been performed on the data presented.

Specific comments:
1) In Line 61, authors incorrectly state that adipose is the “largest endocrine organ by mass”. Muscle, the largest organ by mass, also secretes a vast array of bioactive molecules including myokines and cytokines. This statement should be corrected.

2) The authors use centrifugal separation to differentiate molecules above 300kDa from those under 300kDa, and conclude that CTRP3 measurements in these two fractions correspond to HMW and MMW CTRP3 populations. However, there is the possibility that the <300kDa fraction contains both LMW and MMW species, and the authors do not demonstrate experimentally that MMW CTRP3 is the only CTRP3 oligomeric species in this fraction.

Although the authors cite a previous publication that reported no detectable LMW species in plasma, it should not be assumed that this is the case in a pediatric population. Authors should demonstrate that CTRP3 is not detected (by immunoblot or ELISA) in a <50kDa fraction.

3) Authors should include a schematic or table to summarize the direction of correlations between the measured parameters and HMW or MMW CTRP3 and total CTRP3. This will help the reader better visualize these patterns between the different CTRP3 oligomeric forms.
