# Supplementary material for: CTRP3 and serum triglycerides in children aged 7-10 years
Source: PLoS One. 2020 Dec 3;15(12):e0241813. doi: 10.1371/journal.pone.0241813 (PMC7714231; doi:10.1371/journal.pone.0241813)
Supplement: Attachment — Submitted filename: PONE-D-20-22639_reviewer comments.docx [file pone.0241813.s007.docx]

PLoS ONE reviewer comments

Comments from editor:

(1) Please provide additional details regarding participant consent. In the ethics statement in the Methods and online submission information, please ensure that you have specified what type you obtained (for instance, written or verbal, and if verbal, how it was documented and witnessed). If your study included minors, state whether you obtained consent from parents or guardians. If the need for consent was waived by the ethics committee, please include this information.

- **As requested, we have specified that written consent was obtained from the parents and written assent was obtained from the children.**

(2) Thank you for stating the following in the Funding Section of your manuscript:

Please remove any funding-related text from the manuscript and let us know how you would like to update your Funding Statement. Currently, your Funding Statement reads as follows: [The funders had no role in study design, data collection and analysis, decision to publish, or preparation of the manuscript.]

- **We have removed the reference to our funders within the manuscript and updated the funding statemen as follows:**
- **This work was supported by grants from the Tennessee Board of Regents [Diversity Research Grant TBR E210029] and the National Institute of Diabetes and Digestive and Kidney Diseases [R15 DK114740-01A1]. The funders had no role in study design, data collection and analysis, decision to publish, or preparation of the manuscript.**

(3) Please include your tables as part of your main manuscript and remove the individual files. Please note that supplementary tables (should remain/ be uploaded) as separate "supporting information" files.

- **Table 1 has been added to the main body of the manuscript as requested.**

Comments from reviewer: This study measured CTRP3 HMW and presumed MMW circulating levels from blood samples taken from pediatric donors. Key finding was that circulating triglycerides and VLDL levels negatively correlated with MMW CTRP3, while HMW CTRP3 did not show any statistically significant correlation with the measured parameters.

Significance: The circulating levels of CTRP3 oligomeric complexes have previously been examined in an adult cohort with or without Type 2 Diabetes Mellitus. This is a similar study on a pediatric cohort. While the reported parameters are similar between the two studies, CTRP3 levels have not previously been examined in a pediatric demographic, and this study provides novel information relevant to the growing epidemic of childhood obesity and type 2 diabetes.

The manuscript is clearly written except for some typographical errors throughout the manuscript and a few run-on sentences. Authors should carefully review the manuscript for typographical and grammatical errors. Appropriate statistical analyses have been performed on the data presented.

- **We thank the reviewer for support of our manuscript and suggestions for improvement. We would like to apologize for typographical errors and oversights in the previous version and attest that all authors have carefully reviewed the revised version.**

Specific comments:
1) In Line 61, authors incorrectly state that adipose is the “largest endocrine organ by mass”. Muscle, the largest organ by mass, also secretes a vast array of bioactive molecules including myokines and cytokines. This statement should be corrected.

- **We have removed this statement as the reviewer is correct that in healthy weight persons muscle mass far exceeds adipose tissue.**

2) The authors use centrifugal separation to differentiate molecules above 300kDa from those under 300kDa, and conclude that CTRP3 measurements in these two fractions correspond to HMW and MMW CTRP3 populations. However, there is the possibility that the <300kDa fraction contains both LMW and MMW species, and the authors do not demonstrate experimentally that MMW CTRP3 is the only CTRP3 oligomeric species in this fraction. Although the authors cite a previous publication that reported no detectable LMW species in plasma, it should not be assumed that this is the case in a pediatric population. Authors should demonstrate that CTRP3 is not detected (by immunoblot or ELISA) in a <50kDa fraction.

- **This is an excellent suggestion by the reviewer. We performed additional experiments on these samples to attempt to detect CTRP3 in the <100 kDa fraction by immunoblot. We performed the assay multiple times with three different CTRP3 antibodies and we were unable to detect CTRP3 in the less than 100 kDa fraction (had we been able to detect CTRP3 <100 we would have also examined a <50 kDa fraction).** **We added a representative image as a supplemental file (Supplemental figure 1).**

3) Authors should include a schematic or table to summarize the direction of correlations between the measured parameters and HMW or MMW CTRP3 and total CTRP3. This will help the reader better visualize these patterns between the different CTRP3 oligomeric forms.

- **We appreciate the reviewer’s suggestion and have added the requested information as 3 separate supplemental tables detailing the correlation coefficients between metabolic parameters and total CTRP3 (Table S1), HMW CTRP3 (Table S2), and MMW CTRP3 (Table S3).**
